# 4′-Chloro­biphenyl-4-yl 2,2,2-trichloro­ethyl sulfate

**DOI:** 10.1107/S1600536808038865

**Published:** 2008-11-29

**Authors:** Xueshu Li, Sean Parkin, Larry W. Robertson, Hans-Joachim Lehmler

**Affiliations:** a The University of Iowa, Department of Occupational and Environmental Health, 100 Oakdale Campus, 124 IREH, Iowa City, IA 52242-5000, USA; b University of Kentucky, Department of Chemistry, Lexington, KY 40506-0055, USA

## Abstract

The title compound, C_14_H_10_Cl_4_O_4_S, is an inter­mediate in the synthesis of the PCB sulfate monoester of 4′-chloro-biphenyl-4-ol. Both the sulfate monoester and 4′-chloro-biphenyl-4-ol are metabolites of PCB 3 (4-chloro­biphen­yl). There are two mol­ecules with different conformations in the asymmetric unit. The solid state dihedral angles between the benzene rings are 18.52 (10) and 41.84 (16)° in the two mol­ecules, whereas the dihedral angles between the least-squares plane of the sulfated benzene ring and O—S (Ar—C—O—S) are 66.2 (3) and 89.3 (3)°. The crystal was an inversion twin with a refined component fraction of 0.44 (7).

## Related literature

For similar structures of hydroxy­lated chloro­biphenyls and their derivatives, see: Rissanen *et al.* (1988*a* ▶,*b* ▶); Lehmler *et al.* (2001 ▶, 2002 ▶); Desiraju *et al.* (1979 ▶); Vyas *et al.* (2006 ▶). For a review of structures of sulfuric acid aryl mono esters, see: Brandao *et al.* (2005 ▶). For additional background, see: Letcher *et al.* (2000 ▶); Liu *et al.* (2004 ▶, 2006 ▶); Sacco & James (2005 ▶); Shaikh *et al.* (2008 ▶); Tampal *et al.* (2002 ▶); Hansen (1999 ▶); Robertson & Hansen (2001 ▶).
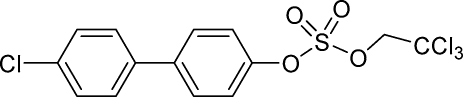


## Experimental

### 

#### Crystal data

C_14_H_10_Cl_4_O_4_S
*M* *_r_* = 416.08
Orthorhombic, 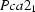

*a* = 9.6305 (19) Å
*b* = 30.273 (6) Å
*c* = 11.330 (2) Å
*V* = 3303.3 (11) Å^3^
*Z* = 8
Mo *K*α radiation
μ = 0.86 mm^−1^
*T* = 90.0 (2) K
0.40 × 0.34 × 0.18 mm

#### Data collection

Nonius KappaCCD diffractometer
Absorption correction: multi-scan (*SCALEPACK*; Otwinowski & Minor, 1997 ▶) *T*_min_ = 0.679, *T*_max_ = 0.861
25566 measured reflections
6476 independent reflections
4862 reflections with *I* > 2σ(*I*)
*R*_int_ = 0.063

#### Refinement

*R*[*F* ^2^ > 2σ(*F* ^2^)] = 0.044
*wR*(*F* ^2^) = 0.109
*S* = 1.05
6476 reflections
415 parameters
1 restraint
H-atom parameters constrained
Δρ_max_ = 0.51 e Å^−3^
Δρ_min_ = −0.56 e Å^−3^
Absolute structure: Flack (1983 ▶), 2481 Friedel Pairs
Flack parameter: 0.44 (7)

### 

Data collection: *COLLECT* (Nonius, 1998 ▶); cell refinement: *SCALEPACK* (Otwinowski & Minor, 1997 ▶); data reduction: *DENZO-SMN* (Otwinowski & Minor, 1997 ▶); program(s) used to solve structure: *SHELXS97* (Sheldrick, 2008 ▶); program(s) used to refine structure: *SHELXL97* (Sheldrick, 2008 ▶); molecular graphics: *XP* in *SHELXTL* (Sheldrick, 2008 ▶); software used to prepare material for publication: *SHELXL97* and local procedures.

## Supplementary Material

Crystal structure: contains datablocks I, global. DOI: 10.1107/S1600536808038865/dn2403sup1.cif

Structure factors: contains datablocks I. DOI: 10.1107/S1600536808038865/dn2403Isup2.hkl

Additional supplementary materials:  crystallographic information; 3D view; checkCIF report

## Figures and Tables

**Fig. 1.**
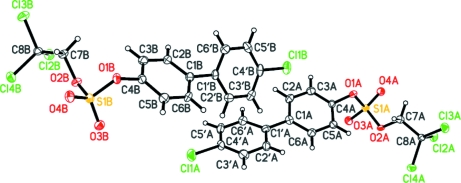
View of the title compound showing the atom-labelling scheme. Displacement ellipsoids are drawn at the 50% probability level.

## supplementary crystallographic information

### Comment

Polychlorinated biphenyls (PCBs) are a major class of man-made, persistent organic pollutants and represent an environmental and health concern due to their toxicity and resistance to biodegradation (Robertson & Hansen, 2001; Hansen, 1999). PCB congeners with a lower degree of chlorination are especially prone to undergo oxidative metabolism to hydroxylated (OH)-PCBs (Letcher *et al.*, 2000). OH-PCBs can be further transformed to glucuronides (Tampal *et al.*, 2002) or sulfates (Liu *et al.*, 2006, Sacco & James, 2005). These PCB metabolites are more hydrophilic than PCBs and OH-PCBs and are expected to be more easily excreted. Despite the potential importance of sulfated PCB metabolites, PCB sulfate monoesters and analogous compounds have not been synthesized experimentally and their detailed molecular structure is unknown. Similarly, only few structures of hydroxylated chlorobiphenyl derivatives (Rissanen *et al.* 1988*a*, 1988*b*; Lehmler *et al.*, 2001, 2002; Desiraju *et al.*, 1979; Vyas *et al.*, 2006) and sulfuric acid aryl mono esters (Brandao *et al.*, 2005) have been reported.

Herein we report the crystal structure of the title compound, a trichloro-ethyl PCB sulfate diester intermediate of a putative sulfate metabolite of PCB3 (4-chlorobiphenyl). The asymmetric unit of the crystal structure contains two molecules with different conformations (Fig. 1), an observation that highlights the flexibility of PCB derivatives that lack multiple *ortho* chlorine substituents. The dihedral angles between the two benzene rings in the biphenyl moiety are 18.52 (10)° and 41.84 (16)° for molecules A and B, respectively. Similar to molecule B, other PCB derivatives with no *ortho* chlorine substituent adopt dihedral angles of 39.42° (4,4'-dichhlorobiphenyl), 41.31 (07)° (3,3',5'-trichloro-4-methoxy-biphenyl) and 43.94 (06)° (3,3',4,4'-tetrachlorobiphenyl) in the solid state (Shaikh *et al.*, 2008). The calculated dihedral angle of the title compound is 41.2° (Shaikh *et al.*, 2008), which is comparable to that of molecule B, but significantly larger than that of molecule A. The dihedral angle formed by the least-squares plane of the sulfated benzene ring and O1—S1 (Ar—C4—O1—S1) was 66.2 (3)° and 89.3 (3)° for molecules A and B, respectively. These dihedral angles are larger than the calculated Ar—C4—O1—S1 dihedral angle of approximately 54° (calculated with AM1 as implemented by ArgusLab, Version 4.0.1). Overall, these deviations from the energetically most favorable conformation of the title compound are due to crystal packing effects, which allow the molecule to adopt an energetically unfavorable conformation to maximize intermolecular interactions, and thus the lattice energy in the crystal.

### Experimental

The title compound was synthesized from 4-chlorobiphenyl-4-ol by sulfation with 2,2,2-trichloroethyl sulfonyl chloride using 4-dimethylaminopyridine as catalyst (Liu *et al.* 2004). Crystals of the title compound suitable for crystal structure analysis were obtained from a methanolic solution by slowly evaporating the solvent.

### Refinement

H atoms were found in difference Fourier maps and subsequently placed in idealized positions with constrained C—H distances of 0.99 Å (CH_2_) and 0.95 Å (C_Ar_H) with *U*_iso_(H) values set to 1.2*U*_eq_ of the attached C atom.

The crystal was an inversion twin with a refined component fraction of 0.44 (7), *i.e.* essentially equal amounts of each component.

### Crystal data

**Table:**

C_14_H_10_Cl_4_O_4_S	*F*_000_ = 1680
*M**_r_* = 416.08	*D*_x_ = 1.673 Mg m^−^^3^
Orthorhombic, *P**c**a*2_1_	Mo *K*α radiation λ = 0.71073 Å
Hall symbol: P 2c -2ac	Cell parameters from 4257 reflections
*a* = 9.6305 (19) Å	θ = 1.0–27.5º
*b* = 30.273 (6) Å	µ = 0.86 mm^−^^1^
*c* = 11.330 (2) Å	*T* = 90.0 (2) K
*V* = 3303.3 (11) Å^3^	Block, colourless
*Z* = 8	0.40 × 0.34 × 0.18 mm

### Data collection

**Table:**

Nonius KappaCCD diffractometer	6476 independent reflections
Radiation source: fine-focus sealed tube	4862 reflections with *I* > 2σ(*I*)
Monochromator: graphite	*R*_int_ = 0.063
Detector resolution: 18 pixels mm^-1^	θ_max_ = 27.5º
*T* = 90.0(2) K	θ_min_ = 2.2º
ω scans at fixed χ = 55°	*h* = −12→12
Absorption correction: multi-scan(SCALEPACK; Otwinowski & Minor, 1997)	*k* = −39→38
*T*_min_ = 0.679, *T*_max_ = 0.861	*l* = −11→14
25566 measured reflections	

### Refinement

**Table:**

Refinement on *F*^2^	Hydrogen site location: inferred from neighbouring sites
Least-squares matrix: full	H-atom parameters constrained
*R*[*F*^2^ > 2σ(*F*^2^)] = 0.044	*w* = 1/[σ^2^(*F*_o_^2^) + (0.0569*P*)^2^] where *P* = (*F*_o_^2^ + 2*F*_c_^2^)/3
*wR*(*F*^2^) = 0.109	(Δ/σ)_max_ = 0.001
*S* = 1.05	Δρ_max_ = 0.51 e Å^−^^3^
6476 reflections	Δρ_min_ = −0.56 e Å^−^^3^
415 parameters	Extinction correction: none
1 restraint	Absolute structure: Flack (1983), 2481 Friedel Pairs
Primary atom site location: structure-invariant direct methods	Flack parameter: 0.44 (7)
Secondary atom site location: difference Fourier map	

### Special details

**Table:**

Geometry. All e.s.d.'s (except the e.s.d. in the dihedral angle between two l.s. planes) are estimated using the full covariance matrix. The cell e.s.d.'s are taken into account individually in the estimation of e.s.d.'s in distances, angles and torsion angles; correlations between e.s.d.'s in cell parameters are only used when they are defined by crystal symmetry. An approximate (isotropic) treatment of cell e.s.d.'s is used for estimating e.s.d.'s involving l.s. planes.
Refinement. Refinement of *F*^2^ against ALL reflections. The weighted *R*-factor *wR* and goodness of fit *S* are based on *F*^2^, conventional *R*-factors *R* are based on *F*, with *F* set to zero for negative *F*^2^. The threshold expression of *F*^2^ > 2σ(*F*^2^) is used only for calculating *R*-factors(gt) *etc*. and is not relevant to the choice of reflections for refinement. *R*-factors based on *F*^2^ are statistically about twice as large as those based on *F*, and *R*- factors based on ALL data will be even larger.

### Fractional atomic coordinates and isotropic or equivalent isotropic displacement parameters (Å^2^)

**Table:**

	*x*	*y*	*z*	*U*_iso_*/*U*_eq_	
S1A	0.37972 (11)	0.52517 (4)	0.22349 (11)	0.0182 (3)	
O1A	0.4951 (3)	0.55663 (9)	0.2732 (3)	0.0189 (7)	
O2A	0.3525 (3)	0.49236 (9)	0.3284 (3)	0.0162 (7)	
O3A	0.2535 (3)	0.54799 (8)	0.2091 (3)	0.0228 (7)	
O4A	0.4438 (3)	0.50234 (10)	0.1291 (3)	0.0219 (7)	
Cl1A	0.32327 (13)	0.81401 (4)	0.83893 (12)	0.0332 (3)	
Cl2A	0.34106 (12)	0.39176 (4)	0.28495 (11)	0.0249 (3)	
Cl3A	0.55811 (11)	0.39177 (4)	0.46162 (11)	0.0250 (3)	
Cl4A	0.28608 (11)	0.42787 (4)	0.51617 (10)	0.0251 (3)	
C1A	0.4100 (4)	0.66199 (14)	0.5036 (4)	0.0185 (10)	
C2A	0.4593 (5)	0.66887 (14)	0.3888 (4)	0.0253 (10)	
H2A	0.4777	0.6981	0.3627	0.030*	
C3A	0.4817 (4)	0.63383 (14)	0.3129 (4)	0.0255 (11)	
H3A	0.5129	0.6390	0.2346	0.031*	
C4A	0.4583 (4)	0.59162 (14)	0.3517 (4)	0.0169 (10)	
C5A	0.4137 (4)	0.58287 (15)	0.4644 (4)	0.0192 (10)	
H5A	0.4002	0.5534	0.4905	0.023*	
C6A	0.3889 (4)	0.61848 (14)	0.5396 (4)	0.0203 (11)	
H6A	0.3567	0.6129	0.6174	0.024*	
C7A	0.4706 (4)	0.46577 (13)	0.3666 (4)	0.0171 (10)	
H7A1	0.5192	0.4804	0.4329	0.021*	
H7A2	0.5371	0.4619	0.3008	0.021*	
C8A	0.4136 (4)	0.42113 (14)	0.4058 (4)	0.0174 (10)	
C1'A	0.3849 (4)	0.69949 (14)	0.5865 (4)	0.0202 (10)	
C2'A	0.3782 (4)	0.69245 (14)	0.7090 (4)	0.0229 (10)	
H2'A	0.3882	0.6634	0.7393	0.028*	
C3'A	0.3573 (5)	0.72738 (14)	0.7862 (4)	0.0278 (11)	
H3'A	0.3519	0.7224	0.8689	0.033*	
C4'A	0.3444 (5)	0.76930 (14)	0.7414 (4)	0.0215 (11)	
C5'A	0.3477 (4)	0.77735 (13)	0.6205 (4)	0.0265 (11)	
H5'A	0.3351	0.8064	0.5905	0.032*	
C6'A	0.3696 (5)	0.74235 (14)	0.5456 (4)	0.0259 (11)	
H6'A	0.3744	0.7477	0.4631	0.031*	
S1B	0.36151 (11)	0.02678 (3)	0.20279 (10)	0.0170 (2)	
O1B	0.4725 (3)	0.06109 (9)	0.1597 (3)	0.0197 (7)	
O2B	0.3431 (3)	−0.00442 (9)	0.0931 (3)	0.0180 (7)	
O3B	0.2310 (3)	0.04755 (8)	0.2178 (3)	0.0214 (7)	
O4B	0.4277 (3)	0.00352 (10)	0.2956 (3)	0.0210 (7)	
Cl1B	0.29897 (13)	0.31088 (4)	−0.42521 (12)	0.0308 (3)	
Cl2B	0.30154 (11)	−0.06864 (4)	−0.10670 (11)	0.0226 (3)	
Cl3B	0.55924 (11)	−0.10661 (4)	−0.02190 (12)	0.0258 (3)	
Cl4B	0.31521 (12)	−0.10380 (4)	0.12863 (11)	0.0249 (3)	
C1B	0.3812 (5)	0.16175 (14)	−0.0805 (4)	0.0198 (10)	
C2B	0.4206 (4)	0.12024 (14)	−0.1204 (4)	0.0201 (10)	
H2B	0.4293	0.1149	−0.2027	0.024*	
C3B	0.4471 (4)	0.08652 (15)	−0.0409 (5)	0.0223 (11)	
H3B	0.4745	0.0581	−0.0680	0.027*	
C4B	0.4335 (4)	0.09461 (14)	0.0765 (5)	0.0183 (10)	
C5B	0.3896 (4)	0.13457 (13)	0.1210 (4)	0.0225 (10)	
H5B	0.3788	0.1390	0.2035	0.027*	
C6B	0.3617 (5)	0.16827 (13)	0.0406 (4)	0.0218 (10)	
H6B	0.3291	0.1960	0.0684	0.026*	
C7B	0.4627 (4)	−0.03186 (14)	0.0616 (4)	0.0182 (10)	
H7B1	0.5231	−0.0362	0.1313	0.022*	
H7B2	0.5177	−0.0173	−0.0011	0.022*	
C8B	0.4097 (4)	−0.07557 (14)	0.0186 (4)	0.0171 (10)	
C1'B	0.3608 (4)	0.19884 (14)	−0.1657 (4)	0.0189 (10)	
C2'B	0.2482 (5)	0.22865 (12)	−0.1553 (4)	0.0239 (10)	
H2'B	0.1834	0.2250	−0.0928	0.029*	
C3'B	0.2307 (5)	0.26276 (13)	−0.2336 (4)	0.0236 (10)	
H3'B	0.1553	0.2827	−0.2245	0.028*	
C4'B	0.3228 (5)	0.26804 (14)	−0.3252 (4)	0.0226 (11)	
C5'B	0.4350 (5)	0.23942 (14)	−0.3388 (4)	0.0247 (11)	
H5'B	0.4992	0.2435	−0.4016	0.030*	
C6'B	0.4515 (5)	0.20517 (14)	−0.2599 (4)	0.0243 (10)	
H6'B	0.5270	0.1853	−0.2700	0.029*	

### Atomic displacement parameters (Å^2^)

**Table:**

	*U*^11^	*U*^22^	*U*^33^	*U*^12^	*U*^13^	*U*^23^
S1A	0.0188 (5)	0.0180 (6)	0.0177 (6)	−0.0007 (4)	0.0011 (5)	0.0019 (5)
O1A	0.0170 (15)	0.0174 (15)	0.0223 (18)	−0.0042 (12)	0.0023 (14)	0.0009 (14)
O2A	0.0143 (14)	0.0185 (15)	0.0160 (18)	−0.0006 (12)	0.0034 (14)	0.0059 (14)
O3A	0.0188 (15)	0.0225 (15)	0.0270 (19)	0.0033 (14)	−0.0026 (15)	0.0035 (15)
O4A	0.0294 (17)	0.0197 (16)	0.0167 (18)	0.0004 (14)	0.0044 (14)	−0.0007 (14)
Cl1A	0.0430 (7)	0.0242 (6)	0.0323 (7)	−0.0018 (5)	−0.0001 (6)	−0.0100 (6)
Cl2A	0.0281 (6)	0.0220 (6)	0.0245 (7)	−0.0004 (5)	−0.0071 (5)	−0.0060 (5)
Cl3A	0.0226 (6)	0.0234 (6)	0.0289 (7)	0.0048 (5)	−0.0031 (5)	0.0023 (5)
Cl4A	0.0253 (6)	0.0273 (6)	0.0227 (6)	0.0025 (5)	0.0079 (5)	0.0047 (5)
C1A	0.019 (2)	0.020 (2)	0.016 (2)	0.0015 (18)	−0.003 (2)	0.000 (2)
C2A	0.031 (3)	0.018 (2)	0.027 (3)	−0.001 (2)	0.002 (2)	0.007 (2)
C3A	0.029 (3)	0.026 (2)	0.022 (3)	0.001 (2)	0.006 (2)	0.005 (2)
C4A	0.015 (2)	0.013 (2)	0.023 (3)	0.0012 (17)	−0.001 (2)	−0.0001 (19)
C5A	0.022 (2)	0.020 (2)	0.016 (2)	0.0024 (18)	0.002 (2)	0.002 (2)
C6A	0.017 (2)	0.019 (2)	0.024 (3)	−0.0005 (19)	−0.002 (2)	0.002 (2)
C7A	0.017 (2)	0.020 (2)	0.014 (2)	0.0019 (19)	−0.0016 (19)	0.002 (2)
C8A	0.014 (2)	0.018 (2)	0.020 (3)	0.0034 (18)	0.002 (2)	0.003 (2)
C1'A	0.019 (2)	0.020 (2)	0.021 (3)	−0.0009 (19)	0.001 (2)	0.005 (2)
C2'A	0.030 (3)	0.016 (2)	0.023 (2)	0.0041 (19)	0.003 (2)	0.001 (2)
C3'A	0.030 (3)	0.028 (3)	0.026 (3)	0.004 (2)	0.001 (2)	0.001 (2)
C4'A	0.022 (2)	0.019 (2)	0.023 (3)	−0.0030 (18)	−0.004 (2)	−0.010 (2)
C5'A	0.030 (3)	0.015 (2)	0.035 (3)	−0.0022 (19)	0.000 (2)	0.000 (2)
C6'A	0.028 (3)	0.020 (2)	0.029 (3)	0.001 (2)	0.003 (2)	0.001 (2)
S1B	0.0177 (5)	0.0161 (5)	0.0173 (6)	−0.0012 (4)	−0.0013 (5)	0.0001 (5)
O1B	0.0210 (15)	0.0143 (15)	0.0237 (19)	−0.0025 (13)	−0.0017 (14)	0.0024 (14)
O2B	0.0156 (15)	0.0191 (16)	0.0192 (19)	0.0025 (12)	−0.0009 (15)	−0.0058 (14)
O3B	0.0179 (15)	0.0198 (15)	0.0265 (18)	0.0001 (13)	−0.0005 (15)	−0.0040 (15)
O4B	0.0246 (16)	0.0219 (17)	0.0166 (18)	0.0001 (14)	−0.0045 (14)	0.0031 (15)
Cl1B	0.0450 (8)	0.0191 (6)	0.0285 (7)	−0.0008 (5)	−0.0068 (6)	0.0054 (5)
Cl2B	0.0221 (5)	0.0255 (6)	0.0203 (6)	−0.0011 (5)	−0.0054 (5)	−0.0012 (5)
Cl3B	0.0193 (6)	0.0255 (6)	0.0326 (7)	0.0056 (5)	−0.0011 (5)	−0.0071 (6)
Cl4B	0.0291 (6)	0.0221 (6)	0.0235 (6)	−0.0017 (5)	0.0031 (5)	0.0044 (5)
C1B	0.021 (2)	0.014 (2)	0.024 (3)	−0.0032 (18)	0.002 (2)	−0.003 (2)
C2B	0.022 (2)	0.016 (2)	0.022 (3)	−0.0002 (19)	0.002 (2)	−0.003 (2)
C3B	0.016 (2)	0.020 (2)	0.031 (3)	0.0001 (18)	0.002 (2)	−0.003 (2)
C4B	0.015 (2)	0.016 (2)	0.024 (3)	0.0002 (18)	−0.003 (2)	0.004 (2)
C5B	0.026 (2)	0.022 (2)	0.020 (2)	−0.0012 (19)	0.001 (2)	−0.003 (2)
C6B	0.026 (2)	0.011 (2)	0.028 (3)	0.0037 (19)	0.003 (2)	−0.0006 (19)
C7B	0.015 (2)	0.018 (2)	0.022 (3)	0.0026 (19)	−0.002 (2)	−0.004 (2)
C8B	0.015 (2)	0.018 (2)	0.019 (2)	0.0035 (18)	0.001 (2)	0.004 (2)
C1'B	0.024 (2)	0.014 (2)	0.019 (3)	−0.0047 (18)	−0.002 (2)	−0.002 (2)
C2'B	0.021 (2)	0.022 (2)	0.029 (3)	−0.0005 (19)	0.005 (2)	−0.002 (2)
C3'B	0.029 (3)	0.015 (2)	0.026 (3)	0.0048 (19)	−0.003 (2)	−0.0025 (19)
C4'B	0.030 (3)	0.014 (2)	0.024 (3)	−0.0054 (19)	−0.013 (2)	0.000 (2)
C5'B	0.029 (3)	0.026 (2)	0.019 (2)	−0.011 (2)	0.001 (2)	0.002 (2)
C6'B	0.026 (2)	0.020 (2)	0.027 (3)	−0.001 (2)	0.003 (2)	−0.005 (2)

### Geometric parameters (Å, °)

**Table:**

S1A—O3A	1.408 (3)	S1B—O3B	1.415 (3)
S1A—O4A	1.415 (3)	S1B—O4B	1.417 (3)
S1A—O1A	1.568 (3)	S1B—O1B	1.568 (3)
S1A—O2A	1.571 (3)	S1B—O2B	1.571 (3)
O1A—C4A	1.427 (5)	O1B—C4B	1.435 (5)
O2A—C7A	1.459 (5)	O2B—C7B	1.464 (5)
Cl1A—C4'A	1.759 (5)	Cl1B—C4'B	1.738 (5)
Cl2A—C8A	1.776 (5)	Cl2B—C8B	1.773 (5)
Cl3A—C8A	1.768 (4)	Cl3B—C8B	1.780 (4)
Cl4A—C8A	1.765 (5)	Cl4B—C8B	1.764 (5)
C1A—C6A	1.394 (6)	C1B—C2B	1.388 (6)
C1A—C2A	1.400 (7)	C1B—C6B	1.399 (6)
C1A—C1'A	1.493 (6)	C1B—C1'B	1.494 (6)
C2A—C3A	1.383 (6)	C2B—C3B	1.386 (6)
C2A—H2A	0.9500	C2B—H2B	0.9500
C3A—C4A	1.370 (6)	C3B—C4B	1.358 (7)
C3A—H3A	0.9500	C3B—H3B	0.9500
C4A—C5A	1.373 (7)	C4B—C5B	1.377 (6)
C5A—C6A	1.395 (6)	C5B—C6B	1.394 (6)
C5A—H5A	0.9500	C5B—H5B	0.9500
C6A—H6A	0.9500	C6B—H6B	0.9500
C7A—C8A	1.524 (6)	C7B—C8B	1.499 (6)
C7A—H7A1	0.9900	C7B—H7B1	0.9900
C7A—H7A2	0.9900	C7B—H7B2	0.9900
C1'A—C6'A	1.386 (6)	C1'B—C6'B	1.392 (6)
C1'A—C2'A	1.406 (7)	C1'B—C2'B	1.416 (6)
C2'A—C3'A	1.387 (6)	C2'B—C3'B	1.371 (6)
C2'A—H2'A	0.9500	C2'B—H2'B	0.9500
C3'A—C4'A	1.372 (6)	C3'B—C4'B	1.375 (7)
C3'A—H3'A	0.9500	C3'B—H3'B	0.9500
C4'A—C5'A	1.392 (7)	C4'B—C5'B	1.394 (6)
C5'A—C6'A	1.374 (6)	C5'B—C6'B	1.379 (6)
C5'A—H5'A	0.9500	C5'B—H5'B	0.9500
C6'A—H6'A	0.9500	C6'B—H6'B	0.9500
			
O3A—S1A—O4A	121.9 (2)	O3B—S1B—O4B	122.1 (2)
O3A—S1A—O1A	110.81 (17)	O3B—S1B—O1B	110.36 (16)
O4A—S1A—O1A	105.03 (18)	O4B—S1B—O1B	104.70 (17)
O3A—S1A—O2A	104.68 (17)	O3B—S1B—O2B	105.19 (17)
O4A—S1A—O2A	109.64 (18)	O4B—S1B—O2B	109.83 (18)
O1A—S1A—O2A	103.31 (17)	O1B—S1B—O2B	103.20 (17)
C4A—O1A—S1A	119.9 (3)	C4B—O1B—S1B	119.7 (3)
C7A—O2A—S1A	116.3 (2)	C7B—O2B—S1B	116.5 (3)
C6A—C1A—C2A	117.5 (4)	C2B—C1B—C6B	118.9 (4)
C6A—C1A—C1'A	120.7 (4)	C2B—C1B—C1'B	120.4 (4)
C2A—C1A—C1'A	121.7 (4)	C6B—C1B—C1'B	120.7 (4)
C3A—C2A—C1A	121.1 (4)	C3B—C2B—C1B	120.4 (5)
C3A—C2A—H2A	119.4	C3B—C2B—H2B	119.8
C1A—C2A—H2A	119.4	C1B—C2B—H2B	119.8
C4A—C3A—C2A	119.3 (4)	C4B—C3B—C2B	119.1 (5)
C4A—C3A—H3A	120.3	C4B—C3B—H3B	120.5
C2A—C3A—H3A	120.3	C2B—C3B—H3B	120.5
C3A—C4A—C5A	122.0 (4)	C3B—C4B—C5B	123.1 (5)
C3A—C4A—O1A	116.8 (4)	C3B—C4B—O1B	119.4 (4)
C5A—C4A—O1A	120.9 (4)	C5B—C4B—O1B	117.4 (4)
C4A—C5A—C6A	118.2 (4)	C4B—C5B—C6B	117.6 (4)
C4A—C5A—H5A	120.9	C4B—C5B—H5B	121.2
C6A—C5A—H5A	120.9	C6B—C5B—H5B	121.2
C1A—C6A—C5A	121.8 (4)	C5B—C6B—C1B	120.8 (4)
C1A—C6A—H6A	119.1	C5B—C6B—H6B	119.6
C5A—C6A—H6A	119.1	C1B—C6B—H6B	119.6
O2A—C7A—C8A	107.1 (3)	O2B—C7B—C8B	108.2 (3)
O2A—C7A—H7A1	110.3	O2B—C7B—H7B1	110.1
C8A—C7A—H7A1	110.3	C8B—C7B—H7B1	110.1
O2A—C7A—H7A2	110.3	O2B—C7B—H7B2	110.1
C8A—C7A—H7A2	110.3	C8B—C7B—H7B2	110.1
H7A1—C7A—H7A2	108.5	H7B1—C7B—H7B2	108.4
C7A—C8A—Cl4A	110.8 (3)	C7B—C8B—Cl4B	111.9 (3)
C7A—C8A—Cl3A	105.5 (3)	C7B—C8B—Cl2B	110.8 (3)
Cl4A—C8A—Cl3A	110.6 (3)	Cl4B—C8B—Cl2B	108.7 (2)
C7A—C8A—Cl2A	111.2 (3)	C7B—C8B—Cl3B	105.9 (3)
Cl4A—C8A—Cl2A	109.3 (2)	Cl4B—C8B—Cl3B	110.1 (2)
Cl3A—C8A—Cl2A	109.5 (2)	Cl2B—C8B—Cl3B	109.4 (3)
C6'A—C1'A—C2'A	117.9 (4)	C6'B—C1'B—C2'B	117.2 (4)
C6'A—C1'A—C1A	121.3 (4)	C6'B—C1'B—C1B	121.1 (4)
C2'A—C1'A—C1A	120.9 (4)	C2'B—C1'B—C1B	121.7 (4)
C3'A—C2'A—C1'A	120.9 (4)	C3'B—C2'B—C1'B	121.3 (4)
C3'A—C2'A—H2'A	119.5	C3'B—C2'B—H2'B	119.4
C1'A—C2'A—H2'A	119.5	C1'B—C2'B—H2'B	119.4
C4'A—C3'A—C2'A	119.0 (4)	C2'B—C3'B—C4'B	119.8 (4)
C4'A—C3'A—H3'A	120.5	C2'B—C3'B—H3'B	120.1
C2'A—C3'A—H3'A	120.5	C4'B—C3'B—H3'B	120.1
C3'A—C4'A—C5'A	121.6 (4)	C3'B—C4'B—C5'B	120.8 (4)
C3'A—C4'A—Cl1A	119.3 (4)	C3'B—C4'B—Cl1B	119.6 (4)
C5'A—C4'A—Cl1A	119.1 (4)	C5'B—C4'B—Cl1B	119.6 (4)
C6'A—C5'A—C4'A	118.5 (4)	C6'B—C5'B—C4'B	119.0 (4)
C6'A—C5'A—H5'A	120.8	C6'B—C5'B—H5'B	120.5
C4'A—C5'A—H5'A	120.8	C4'B—C5'B—H5'B	120.5
C5'A—C6'A—C1'A	122.1 (5)	C5'B—C6'B—C1'B	121.9 (4)
C5'A—C6'A—H6'A	118.9	C5'B—C6'B—H6'B	119.0
C1'A—C6'A—H6'A	118.9	C1'B—C6'B—H6'B	119.0
			
O3A—S1A—O1A—C4A	32.4 (4)	O3B—S1B—O1B—C4B	−38.3 (4)
O4A—S1A—O1A—C4A	165.9 (3)	O4B—S1B—O1B—C4B	−171.3 (3)
O2A—S1A—O1A—C4A	−79.2 (3)	O2B—S1B—O1B—C4B	73.7 (3)
O3A—S1A—O2A—C7A	−176.8 (3)	O3B—S1B—O2B—C7B	−177.1 (3)
O4A—S1A—O2A—C7A	50.8 (3)	O4B—S1B—O2B—C7B	−44.0 (4)
O1A—S1A—O2A—C7A	−60.8 (3)	O1B—S1B—O2B—C7B	67.2 (3)
C6A—C1A—C2A—C3A	2.1 (7)	C6B—C1B—C2B—C3B	−3.4 (7)
C1'A—C1A—C2A—C3A	−179.8 (4)	C1'B—C1B—C2B—C3B	176.3 (4)
C1A—C2A—C3A—C4A	−1.6 (7)	C1B—C2B—C3B—C4B	0.3 (6)
C2A—C3A—C4A—C5A	−0.3 (7)	C2B—C3B—C4B—C5B	2.3 (7)
C2A—C3A—C4A—O1A	−174.5 (4)	C2B—C3B—C4B—O1B	−174.6 (4)
S1A—O1A—C4A—C3A	−116.3 (4)	S1B—O1B—C4B—C3B	−90.7 (4)
S1A—O1A—C4A—C5A	69.5 (5)	S1B—O1B—C4B—C5B	92.3 (4)
C3A—C4A—C5A—C6A	1.6 (7)	C3B—C4B—C5B—C6B	−1.6 (7)
O1A—C4A—C5A—C6A	175.5 (4)	O1B—C4B—C5B—C6B	175.3 (4)
C2A—C1A—C6A—C5A	−0.8 (6)	C4B—C5B—C6B—C1B	−1.6 (7)
C1'A—C1A—C6A—C5A	−179.0 (4)	C2B—C1B—C6B—C5B	4.0 (7)
C4A—C5A—C6A—C1A	−1.0 (7)	C1'B—C1B—C6B—C5B	−175.7 (4)
S1A—O2A—C7A—C8A	−146.0 (3)	S1B—O2B—C7B—C8B	143.8 (3)
O2A—C7A—C8A—Cl4A	−55.9 (4)	O2B—C7B—C8B—Cl4B	−61.5 (4)
O2A—C7A—C8A—Cl3A	−175.6 (3)	O2B—C7B—C8B—Cl2B	59.9 (4)
O2A—C7A—C8A—Cl2A	65.8 (4)	O2B—C7B—C8B—Cl3B	178.4 (3)
C6A—C1A—C1'A—C6'A	−162.8 (4)	C2B—C1B—C1'B—C6'B	−41.0 (6)
C2A—C1A—C1'A—C6'A	19.1 (7)	C6B—C1B—C1'B—C6'B	138.6 (5)
C6A—C1A—C1'A—C2'A	18.2 (6)	C2B—C1B—C1'B—C2'B	138.2 (5)
C2A—C1A—C1'A—C2'A	−159.9 (4)	C6B—C1B—C1'B—C2'B	−42.1 (6)
C6'A—C1'A—C2'A—C3'A	−0.1 (7)	C6'B—C1'B—C2'B—C3'B	−1.1 (7)
C1A—C1'A—C2'A—C3'A	178.9 (4)	C1B—C1'B—C2'B—C3'B	179.6 (4)
C1'A—C2'A—C3'A—C4'A	−0.7 (7)	C1'B—C2'B—C3'B—C4'B	0.9 (7)
C2'A—C3'A—C4'A—C5'A	2.0 (7)	C2'B—C3'B—C4'B—C5'B	−0.8 (7)
C2'A—C3'A—C4'A—Cl1A	−178.0 (3)	C2'B—C3'B—C4'B—Cl1B	179.2 (3)
C3'A—C4'A—C5'A—C6'A	−2.5 (7)	C3'B—C4'B—C5'B—C6'B	0.9 (7)
Cl1A—C4'A—C5'A—C6'A	177.6 (3)	Cl1B—C4'B—C5'B—C6'B	−179.0 (3)
C4'A—C5'A—C6'A—C1'A	1.6 (7)	C4'B—C5'B—C6'B—C1'B	−1.2 (7)
C2'A—C1'A—C6'A—C5'A	−0.4 (7)	C2'B—C1'B—C6'B—C5'B	1.3 (7)
C1A—C1'A—C6'A—C5'A	−179.4 (4)	C1B—C1'B—C6'B—C5'B	−179.5 (4)
